# Transition-transversion encoding and genetic relationship metric in ReliefF feature selection improves pathway enrichment in GWAS

**DOI:** 10.1186/s13040-018-0186-4

**Published:** 2018-11-03

**Authors:** M. Arabnejad, B. A. Dawkins, W. S. Bush, B. C. White, A. R. Harkness, B. A. McKinney

**Affiliations:** 1 Tandy School of Computer Science, The University of Tulsa, 800 S. Tucker Dr, Tulsa, OK 74104 USA; 2 Department of Mathematics, The University of Tulsa, Tulsa, OK 74104 USA; 3 Institute for Computational Biology, Case Western Reserve University, 2103 Cornell Road, Cleveland, OH 44106 USA; 4 Department of Psychology, The University of Tulsa, Tulsa, OK 74104 USA

**Keywords:** Machine learning, Feature selection, Genome-wide association study (GWAS), Genetic relationship matrix (GRM), Transition and transversion

## Abstract

**Background:**

ReliefF is a nearest-neighbor based feature selection algorithm that efficiently detects variants that are important due to statistical interactions or epistasis. For categorical predictors, like genotypes, the standard metric used in ReliefF has been a simple (binary) mismatch difference. In this study, we develop new metrics of varying complexity that incorporate allele sharing, adjustment for allele frequency heterogeneity via the genetic relationship matrix (GRM), and physicochemical differences of variants via a new transition/transversion encoding.

**Methods:**

We introduce a new two-dimensional transition/transversion genotype encoding for ReliefF, and we implement three ReliefF attribute metrics: 1.) genotype mismatch (GM), which is the ReliefF standard, 2.) allele mismatch (AM), which accounts for heterozygous differences and has not been used previously in ReliefF, and 3.) the new transition/transversion metric. We incorporate these attribute metrics into the ReliefF nearest neighbor calculation with a Manhattan metric, and we introduce GRM as a new ReliefF nearest-neighbor metric to adjust for allele frequency heterogeneity.

**Results:**

We apply ReliefF with each metric to a GWAS of major depressive disorder and compare the detection of genes in pathways implicated in depression, including Axon Guidance, Neuronal System, and G Protein-Coupled Receptor Signaling. We also compare with detection by Random Forest and Lasso as well as random/null selection to assess pathway size bias.

**Conclusions:**

Our results suggest that using more genetically motivated encodings, such as transition/transversion, and metrics that adjust for allele frequency heterogeneity, such as GRM, lead to ReliefF attribute scores with improved pathway enrichment.

**Electronic supplementary material:**

The online version of this article (10.1186/s13040-018-0186-4) contains supplementary material, which is available to authorized users.

## Background

ReliefF is a nearest-neighbors feature selection algorithm that is known for its ability to identify statistical interactions in high dimensional data [1, 2]. Specifically, it has been shown to identify gene-gene interaction effects in simulated and real genome-wide association studies (GWAS) [2]. ReliefF uses what is called a “diff” function to determine nearest neighbors in the space of single nucleotide polymorphisms (SNPs) and to compute the importance of a SNP based on its ability to separate cases and controls in the SNP space.

While ReliefF analysis of GWAS data depends critically on its ability to measure the degree of dissimilarity between genotype states, the diff function used up to this point has been extremely simple. For example, the standard ReliefF genotype diff between two subjects is binary: the diff is 0 when the genotypes of the two subjects at a SNP are identical and 1 if their genotypes are not identical. The distance between a pair of subjects is obtained by summing the diff values in a city-block (Manhattan) metric across all SNPs. The binary nature of this standard diff is likely an oversimplification that misses information because there are degrees of difference between genotypes. In addition to a metric based on allele-sharing differences, we develop a transition/transversion (Ti/Tv) metric that accounts for physicochemical differences of nucleotides and a Genetic Relationship Matrix (GRM) [3] metric that accounts for allele frequency heterogeneity.

The main goal of the current study is to develop and compare combinations of metrics between SNPs and subjects in ReliefF feature selection. We also compare with statistical learning feature selection methods Random Forest [4] and Lasso (least absolute shrinkage and selection operator) [5]. Lasso has been used in GWAS [6, 7] but is parametric and generally uses a strong independence assumption among features. Random Forest has also been applied to GWAS and has fewer constraints than regression, which is an advantage when a multivariate genetic architecture may be involved in disease susceptibility [8–10]. We previously used Random Forest and penalized logistic regression as methods for comparing epistasis detection in simulated data [11]. When genes interact and have no marginal effect, we found that Random Forest has limited power to detect gene-gene interactions in high dimensional data, which was confirmed in Ref. [12]. The diminished Random Forest importance scores for interacting variants is attributable to the independence assumption in the tree node-splitting criterion.

In the current study, we use enrichment of pathways related to major depressive disorder (MDD) to compare feature selection methods. Early GWAS studies of MDD had limited success at finding significant variants due to the contribution of many loci with small effect sizes as well as the heterogeneous characteristics of MDD and the complex interaction between genetic variation and environmental factors [13]. In recent studies, many small main effect loci have been identified through the accumulation of extremely large samples [14, 15]. Identifying broad pools of regulating, modulating or interacting SNPs that confer risk for a target disorder is an important goal. For example, bipolar disorder (BD) may occur in a family in which there is a primary susceptibility gene, but the majority of BD may involve the interactions of multiple genes [16]. Detecting these interaction effects with ReliefF may be improved by tailoring metrics to GWAS data.

The current study is organized as follows. We describe the overall strategy, briefly review the relevant components of ReliefF, describe a new allele sharing ReliefF diff, develop a new 2d transition/transversion genotype encoding and accompanying new ReliefF diff. We also introduce the first use of GRM to compute nearest hits and misses in ReliefF. We apply ReliefF using combinations of diffs and metrics with other statistical learning methods to 463 cases of MDD and 459 controls, and we test the top selected SNPs and their corresponding genes for overlap with biological pathways related to mental disorders. Our results suggest that using more genetically motivated metrics (allele sharing and Ti/Tv) and metrics that adjust for allelic heterogeneity (standardization by allele frequency in GRM) lead to ReliefF scores that improve enrichment of biologically relevant pathways.

## Methods

**Fig. 1:**
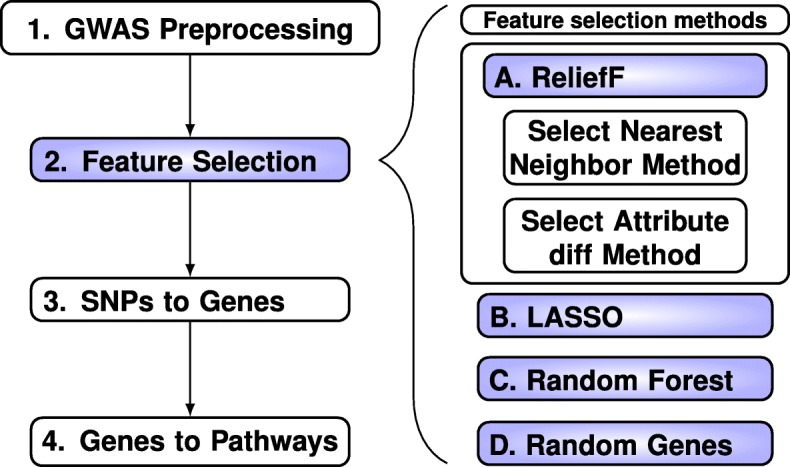
Overall evaluation strategy. (1) Preprocess the GWAS data by minor allele frequency and linkage disequilibrium filtering, (2) Apply feature selection algorithms (purple, right panel A-D), (3) Map top SNPs from methods A-D to gene symbols, and (4) Map genes to the target pathways to compare enrichment. The feature selection algorithms (right panel) are (A) ReliefF with combinations of nearest-neighbor and diff metrics (additional details in Fig. 2), (B) Lasso penalized regression with principal component correction, (C) Random Forest permutation importance, and (D) random sets of genes to assess pathway size bias

The goal of this study is to develop new ReliefF metrics for GWAS and compare them based on their ability to enrich for genes in pathways that have prior evidence for relevance to a phenotype. Our overall strategy (Fig. 1) is to compare enrichment of known relevant pathways. The analysis for each feature selection method involves four steps (left side Fig. 1). First we filter with minor allele threshold 0.01 and linkage-disequilibrium (LD) threshold 0.5 (Step 1, Fig. 1), which results in 281,648 SNPs prior to the application of each method. We choose the top SNPs from each feature selection method (Step 2) including ReliefF (Part A, Fig. 1), Lasso (Part B, Fig. 1), Random Forest (Part C, Fig. 1) and Random Genes of size 500 (Part D, Fig. 1). The purpose of Random Genes is to estimate the effect of pathway size on enrichment due to chance. For each method, we choose the number of tops SNPs so that when we map SNPs to gene symbols (Step 3) we obtain 500 unique genes. Finally, we compare the number of genes detected for each of the biologically relevant pathways (Step 4).

**Fig. 2:**
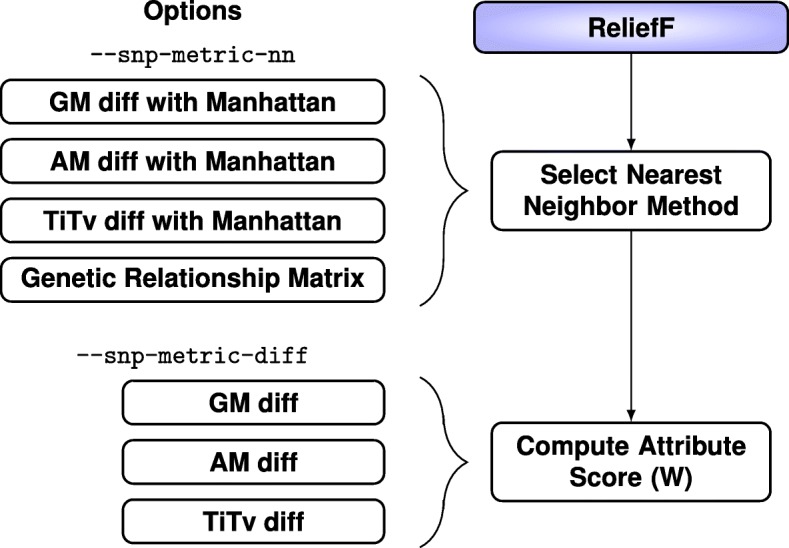
ReliefF combinations of metrics and diffs. We use three methods (two new) for computing the attribute diff value (Eqs. 4, 5, 8): GM, AM, and TiTv (genotype mismatch, allele mismatch, and transition/transversion) with the flag --snp-metric-diff in our inbix software. These three diffs also can be combined with the Manhattan metric (Eq. 9) to create three methods for computing the nearest neighbor distances in the space of all SNPs with the --snp-metric-nn flag combined with --manhattan (Euclidean is also an option but we focus on Manhattan). The fourth nearest neighbor method is the genetic relationship matrix, which does not use the diffs and is called by the grm option with --snp-metric-nn flag. We focus on six combinations of nearest neighbor and diff methods: each of the three diffs with Manhattan for nearest neighbors and each of the three diffs used with GRM to compute nearest neighbors

For ReliefF, we implement four methods for computing the nearest-neighbor distance matrix in our inbix software with --snp-metric-nn flag (Fig. 2) and three diff functions for computing the attribute importance score with --snp-metric-diff (Fig. 2). The three attribute importance diffs incorporate increasing nucleotide information: binary genotype mismatch (GM), allele mismatch (AM), and transition/transversion (Ti/Tv). Each of these diffs can be combined with a Manhattan metric to create three nearest neighbor methods. The Euclidean metric is also an option in our software. The last nearest-neighbor method is based on GRM. Each metric and diff function is discussed in detail below. We focus on six combinations: each of the three diffs used in the Manhattan metric for nearest neighbors and each of the three diffs used with GRM to compute nearest neighbors.

### Relief feature selection algorithm

The goal of the Relief algorithm [17] is to estimate the importance of attributes according to how well their values distinguish between nearest neighbors from different classes (e.g., cases and controls). The Relief algorithm uses a base “diff” function for the distance matrix to compute nearest neighbors, but the diff is also used for computing attribute importance. Recently we reformulated the ReliefF weight in a compact mathematical form as a difference of means between nearest misses and hits [18]. A hit is defined for a given instance or subject R_i_ (*i=1…m*) as another instance that has the same class label (case or control) as that of R_i_, and a miss is another instance with a different class label from R_i_. Once a distance matrix, D, is computed between all instances (discussed more below), the reformulated ReliefF score for SNP *g*_*ν*_ (*ν* =1…N) can be written as:

1 $$ {W}_R\left[{g}_{\nu}\right]={\overline{M}}_{g_{\nu }}-{\overline{H}}_{g_{\nu }}. $$

where the quantities

2 $$ {\overline{M}}_{g_{\nu }}=\frac{1}{mk}\sum \limits_{i=1}^m\sum \limits_{j=1}^k\mathrm{diff}\left({g}_{\nu },{R}_i,{M}_j\left({R}_i\right)\right). $$

and

3 $$ {\overline{H}}_{g_{\nu }}=\frac{1}{mk}\sum \limits_{i=1}^m\sum \limits_{j=1}^k\mathrm{diff}\left({g}_{\nu },{R}_i,{H}_j\left({R}_i\right)\right). $$

are the mean diffs with respect to SNP *g*_*ν*_ of all subjects R_i_ (*i=1…m*) from their *k*-nearest-neighbor misses [M_j_(R_i_) in Eq. (2)] and hits [H_j_(R_i_) in Eq. (3)]. The k nearest misses for a subject R_i_, are the k subjects nearest to R_i_ but in a different phenotype class than R_i_. Similarly, the set of hits of R_i_ is the set of k subjects that are nearest to R_i_ while being in the same phenotype class as subject R_i_. An importance weight of SNP *g*_*ν*_ (W_R_[*g*_*ν*_]) is higher if the average of the miss diffs for the instances is greater than the average hit diffs. Thus, a SNP with a greater positive value of W_R_(i.e,. ${\overline{M}}_{g_{\nu }}>{\overline{H}}_{g_{\nu }}$) is a better predictor of the phenotype because the genotypes of the SNP tend to separate instances in different classes more than instances in the same class. The diff function computes the amount that two genotypes are different for SNP *g*_*ν*_ between two subjects R_i_ and R_j_. In the next subsection, we discuss in detail the new and old diff functions that will be compared.

### New ReliefF diffs and metrics

We introduce three diff functions for measuring the genetic dissimilarity between pairs of individuals at a single locus. The first diff is the standard used in ReliefF for categorical variables, which we refer to as genotype mismatch (GM). The second metric accounts for allele sharing, which we refer to as allele mismatch (AM). The third diff further accounts for mutation type through transition/transversion differences (Ti/Tv). These first three diffs will be used to compute attribute importance and to compute city-block (Manhattan) distances between subjects. We will discuss these nearest-neighbor metrics and the genetic relationship matrix (GRM) in a later subsection.

#### Genotype mismatch

The standard metric used by ReliefF for categorical variables uses a binary mismatch diff. For SNPs, the genotype mismatch (GM) is a 0 or 1 difference between two individuals (*R*_1_, *R*_2_) for a SNP, *g*_*ν*_, based on the individuals’ genotypes. The diff function is

4 $$ {\mathrm{diff}}_{GM}\left({g}_{\nu },{\mathrm{R}}_1,{\mathrm{R}}_2\right)=\left\{\begin{array}{ccc}0&, & \mathrm{genotype}\left({g}_{\nu },{R}_1\right)==\mathrm{genotype}\left({g}_{\nu },{R}_2\right)\\ {}1&, & \mathrm{otherwise}\end{array}\right\} $$

where genotype(*g*_*ν*_, *R*_1_) is the genotype for individual *R*_1_ for SNP *g*_*ν*_. In other words, two individuals have zero diff if they have identical genotypes and they have unit diff if they have different genotypes.

#### Allele mismatch

A potential drawback of GM is that it is not sensitive to heterozygous genotype differences when computing the diff. The following allele mismatch (AM) diff accounts for the difference in the number of alleles for a SNP when computing the difference between two individuals. The difference of two individuals can be calculated by the following formula

5 $$ {\mathrm{diff}}_{AM}\ \left({g}_{\nu },{\mathrm{R}}_1,{\mathrm{R}}_2\right)=\frac{1}{2}\times \mid {g}_{1\nu }-{g}_{2\nu}\mid $$

where *g*_*iν*_ is the number of copies of the reference allele for the *ν*^*th*^ SNP of the *i*^*th*^ individual. In other words, the value of *g*_1*ν*_ is the number of minor alleles in the genotype: 0, 1 or 2. Then the return value of diff_*AM*_ (*g*_*ν*_, R_1_, R_2_) is 0, 0.5 or 1 when the two individual have 2, 1 or 0 alleles in common, respectively.

#### Transition/transversion 2d encoding and associated diff

**Fig. 3:**
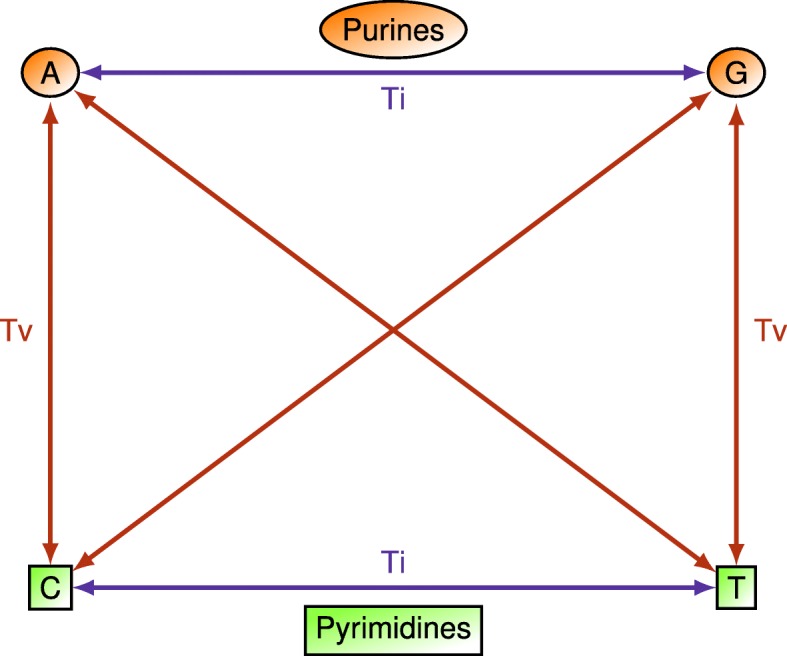
Definition of transitions and transversions. Nucleotides A and G (orange circles) are in the purine family. Nucleotides C and T (green squares) are in the pyrimidine family. When alleles of a genotype are both from the same family (both purine or both pyrimidine), the genotype is called a transition (blue arrows). When alleles of a genotype are each from the opposite family (one purine and pyrimidine), the genotype is called a transversion (red arrows)

The AM diff increases the sensitivity over the GM diff because with AM a heterozygous state is half the distance between either homozygous state. Next our goal is to incorporate additional physicochemical information into the diff based on transition and transversion mutations. A transition is a point mutation (blue arrows in Fig. 3) that changes a purine nucleotide to another purine (A ↔ G) (orange circles in Fig. 3) or a pyrimidine nucleotide to another pyrimidine (C ↔ T) (green squares in Fig. 3). Transversion refers to the substitution of a purine (A or G) for a pyrimidine (C or T) or vice versa (red arrows in Fig. 3) [19]. For the Ti/Tv diff function, we classify genotypes as transitions or transversions and hypothesize that an allele mismatch at a transversion genotype is greater than the same mismatch for a transition genotype.

**Fig. 4:**
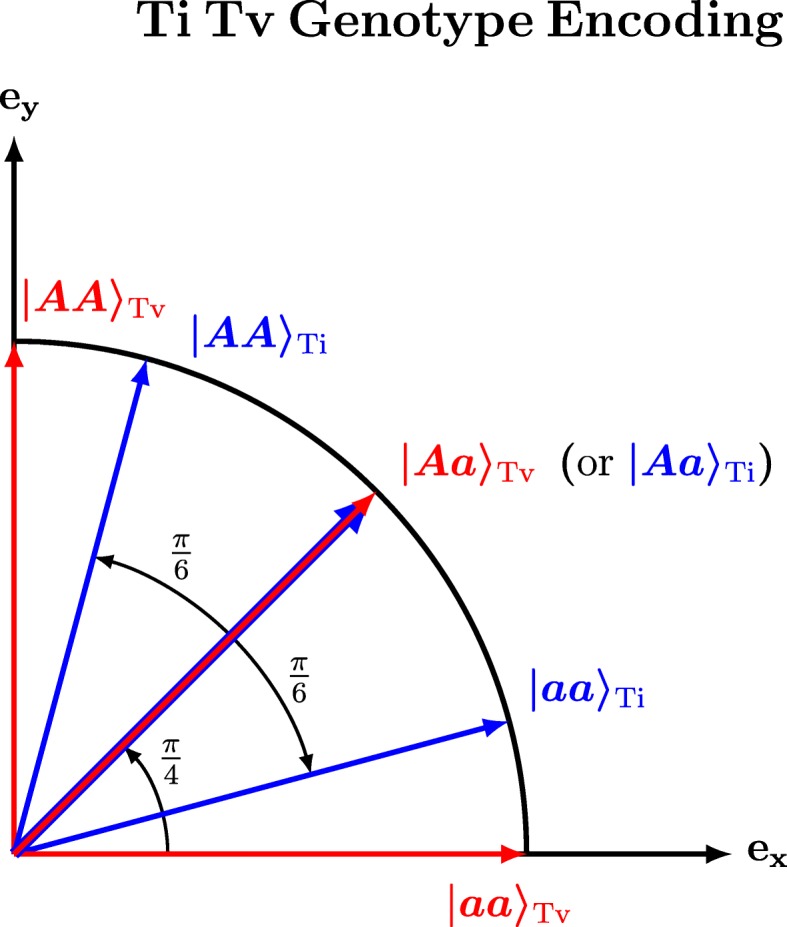
Two-dimensional transition/transversion genotype encoding. Genotypes are represented as vectors on a unit circle and their similarity is the cos^2^*θ* of their angular separation. Transversion genotype vectors (red) are more expanded than transitions (by *π*/4) to account for the alleles (A and a) coming from different nucleotide families (one purine and one pyrimidine). Transition genotype vectors (blue) are more compressed together (by *π*/6) to account for the alleles (A and a) coming from the same nucleotide family (both purine or both pyrimidine). For both transition and transversion SNPs, we center the heterozygous genotype vector at *π*/4

Before constructing the Ti/Tv diff, we first introduce a 2d Ti/Tv genotype encoding (Fig. 4) in which a genotype is a point on a unit circle in the Cartesian plane with basis vectors **e**_*x*_ = (1, 0) and **e**_*y*_ = (0, 1). Below we use Dirac bra-ket notation, where | *x*〉 represents a column vector and 〈*x*| represents a row vector. An example of this encoding that is appropriate for transversion genotypes or an additive encoding (red arrows in Fig. 4) has the two homozygous states orthogonal (*θ* = *π*/2) to each other: | *aa*〉_Tv_ = **e**_*x*_ on the horizontal axis and | *AA*〉_Tv_ = **e**_*y*_ on the vertical axis. And then the heterozygous state is an equal mixture (*θ* = *π*/4) of the homozygous states: $\mid Aa\Big\rangle {}_{\mathrm{Tv}}=\left(\frac{1}{\sqrt{2}},\frac{1}{\sqrt{2}}\right)$.

For transition mutations, we want our encoding to contract the distance (relative to transversion encoding) between two homozygous states and between a homozygous state and the heterozygous state (blue arrows, Fig. 4). Again we let the heterozygous state be $\mid Aa\Big\rangle {}_{\mathrm{Ti}}=\left(\frac{1}{\sqrt{2}},\frac{1}{\sqrt{2}}\right)$, centered on the diagonal (*θ* = *π*/4) like transversions, but instead of being orthogonal, the homozygous states are contracted toward the diagonal with an angle of separation *θ* = *π*/6.

We use | *ψ*_*i*_(*g*_*ν*_)〉 to represent the six possible 2d Ti/Tv genotypes (Fig. 4) for individual R_i_ for SNP *g*_*ν*_. The TiTv similarity between two individuals (R_1_ and R_2_) for SNP *g*_*ν*_ is the squared dot product of the individuals’ Ti/Tv encoding (| *ψ*_1_(*g*_*ν*_)〉 and | *ψ*_2_(*g*_*ν*_)〉):

6 $$ {\mathrm{sim}}_{\mathrm{TiTv}}\left({g}_{\nu },{R}_1,{R}_2\right)={\left|\left\langle {\psi}_{R_1}\left({g}_{\nu}\right)|{\psi}_{R_2}\left({g}_{\nu}\right)\right\rangle \right|}^2={\cos}^2\left({\theta}_{12}\right) $$

where $\left\langle {\psi}_{R_1}\left({g}_{\nu}\right)|{\psi}_{R_2}\left({g}_{\nu}\right)\right\rangle$ is Dirac notation for the dot product of the Ti/Tv genotype vectors and $\Big\langle {\psi}_{R_1}\left({g}_{\nu}\right)\mid$ is the transpose of the column vector $\mid {\psi}_{R_1}\left({g}_{\nu}\right)\Big\rangle$. From Eq. (6), the diff can be written as

7 $$ {\mathrm{diff}}_{\mathrm{TiTv}}\left({g}_{\nu },{R}_1,{R}_2\right)=1-{\mathrm{sim}}_{\mathrm{TiTv}}\left({g}_{\nu },{R}_1,{R}_2\right) $$

or

8 $$ {\mathrm{diff}}_{\mathrm{TiTv}}\left({g}_{\nu },{R}_1,{R}_2\right)=1-{\left|\left\langle {\psi}_{R_1}\left({g}_{\nu}\right)|{\psi}_{R_2}\left({g}_{\nu}\right)\right\rangle \right|}^2 $$

If *g*_*ν*_ is a transversion and individual R_1_ has homozygous genotype $\mid {\psi}_{R_1}\left({g}_{\nu}\right)\left\rangle =\mid AA\right\rangle {}_{\mathrm{Tv}}$ and R_2_ has heterozygous genotype $\mid {\psi}_{R_2}\left({g}_{\nu}\right)\left\rangle =\mid Aa\right\rangle {}_{\mathrm{Tv}}$, then the diff value is

$$ {\mathrm{diff}}_{\mathrm{TiTv}}\left({g}_{\nu}\in \mathrm{Tv},{R}_1,{R}_2\right)=1-{\left|{\left\langle AA| Aa\right\rangle}_{\mathrm{Tv}}\right|}^2=1-{\left|\left(0,1\right)\bullet \left(\begin{array}{c}\frac{1}{\sqrt{2}}\\ {}\frac{1}{\sqrt{2}}\end{array}\right)\right|}^2=1-\frac{1}{2}=\frac{1}{2}. $$

For two individuals at a transversion SNP that are opposite homozygotes ($\mid {\psi}_{R_1}\left({g}_{\nu}\right)\left\rangle =\mid AA\right\rangle {}_{\mathrm{Tv}}$ and $\mid {\psi}_{R_2}\left({g}_{\nu}\right)\left\rangle =\mid aa\right\rangle {}_{\mathrm{Tv}}$):

$$ {\mathrm{diff}}_{\mathrm{TiTv}}\left({g}_{\nu}\in \mathrm{Tv},{R}_1,{R}_2\right)=1-{\left|{\left\langle AA| aa\right\rangle}_{\mathrm{Tv}}\right|}^2=1-{\left|\left(0,1\right)\bullet \left(\begin{array}{c}1\\ {}0\end{array}\right)\right|}^2=1-0=1. $$

Thus, the Ti/Tv diff for transversion SNPs is equivalent to AM because a heterozygous state is half the distance between either homozygous state.

Repeating the above examples for the transition encoding, the diff between $\mid {\psi}_{R_1}\left({g}_{\nu}\right)\left\rangle =\mid AA\right\rangle {}_{\mathrm{Ti}}$ and $\mid {\psi}_{R_2}\left({g}_{\nu}\right)\left\rangle =\mid Aa\right\rangle {}_{\mathrm{Ti}}$ is

$$ {\mathrm{diff}}_{\mathrm{Ti}\mathrm{Tv}}\left({g}_{\nu}\in \mathrm{Ti},{\mathrm{R}}_1,{R}_2\right)=1-{\left|{\left\langle AA| Aa\right\rangle}_{\mathrm{Ti}}\right|}^2=1-{\left|\left(1/2,\sqrt{3}/2\right)\bullet \left(\begin{array}{c}\sqrt{3}/2\\ {}1/2\end{array}\right)\right|}^2=1-\frac{3}{4}=\frac{1}{4}. $$

For individuals that are opposite homozygotes ($\mid {\psi}_{R_1}\left({g}_{\nu}\right)\left\rangle =\mid AA\right\rangle {}_{\mathrm{Ti}}$ and $\mid {\psi}_{R_2}\left({g}_{\nu}\right)\left\rangle =\mid aa\right\rangle {}_{\mathrm{Ti}}$):

$$ {\mathrm{diff}}_{\mathrm{Ti}\mathrm{Tv}}\left({g}_{\nu}\in \mathrm{Ti},{R}_1,{R}_2\right)=1-{\left|{\left\langle AA| aa\right\rangle}_{\mathrm{Ti}}\right|}^2=1-{\left|\left(1/2,\sqrt{3}/2\right)\bullet \left(\begin{array}{c}1\\ {}0\end{array}\right)\right|}^2=1-\frac{1}{4}=\frac{3}{4}. $$

By design, this encoding causes the Ti homozygous diffs (3/4 difference) to be smaller than diffs between Tv homozygous states (1 difference) because transition mutations stay in the same biochemical family (purine to purine or pyrimidine to pyrimidine). Similarly, the encoding causes diffs between heterozygous and homozygous Ti genotypes (1/4 difference) to be smaller than the corresponding Tv diffs (1/2 difference).

We catalog the output of the GM, AM, and Ti/Tv diff functions for all combinations of genotypes and for the cases when the SNP is a transition or transversion (Table 1). The GM diff (green) treats homozygous differences (AA vs aa) the same as a heterozygous difference (AA vs Aa) or (aa vs Aa). The AM diff (orange) is sensitive to allele differences between homozygotes (AA or aa) and heterozygotes (Aa), where the difference is 1/2 of the homozygous difference. However, AM does not distinguish between transition and transversion genotypes. The Ti/Tv diff (blue) is also sensitive to allele differences but further distinguishes between transition and transversion allele changes, treating transition genotypes as more similar than the corresponding transversion genotypes. In this study, we focus on biallelic SNPs. The cases of tri-allelic and copy number variation may be interesting future modifications.

**Table 1.**
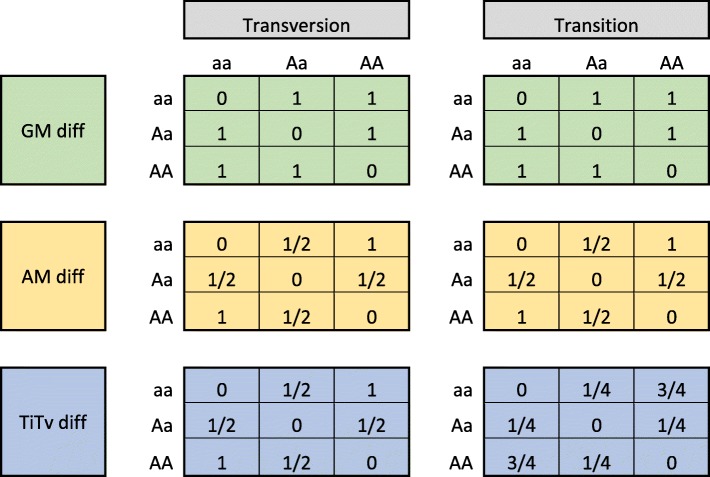
Comparison of the diff(*g*_*ν*_, *R*_*i*_, *R*_*j*_) between individuals *R*_*i*_ and *R*_*j*_ for SNP *g*_*ν*_ for different genotype combinations using GM (green, Eq. 4), AM (orange, Eq. 5), and Ti/Tv (blue, Eq. 8) for all combinations of genotypes and for the cases when the SNP is a transition or transversion

The GM diff (green) treats homozygous differences (AA vs aa) the same as an allele difference (AA vs Aa) or (aa vs Aa). The AM diff (orange) is sensitive to allele differences between homozygotes (AA or aa) and heterozygotes (Aa). The TiTv diff (blue) is sensitive to allele differences but further distinguishes between transition and transversion allele changes, treating transition genotypes as more similar than the corresponding transversion genotypes

#### Nearest-neighbor distances based on Manhattan metric and the genetic relationship matrix (GRM)

We compare the above diffs (GM, AM, Ti/Tv) (Eqs. 4, 5, 8) based on their influence in the attribute importance score (Eqs. 1–3). However, the diff may also be used to determine the distance between subjects by summing the absolute value of the diffs between a pair of subjects R_i_ and R_j_ for all genetic variants *g*_*ν*_ (*ν* = 1…*N*) in a city-block metric (Eq. 9 below). The standard ReliefF nearest-neighbor distance matrix for categorical variables uses the diff=diff_GM_ (Eq. 4) in the following metric:

9 $$ {D}_{ij}^{city}=\sum \limits_{\nu =1}^N\left|\mathrm{diff}\left({g}_{\nu },{R}_i,{R}_j\right)\right|. $$

However, one may also replace diff=diff_AM_ (Eq. 5) or diff=diff_TiTv_ (Eq. 8) in Eq. (9). Regardless of the diff, when an attribute’s importance score is calculated, it uses the k nearest neighbors as determined in the space of all other attributes, which allows ReliefF to identify important attributes that may involve complex higher-order interaction architecture.

We also propose a more sophisticated metric for computing the nearest-neighbor distance matrix based on the Genetic Relationship Matrix (GRM) from GCTA. The GRM is used to calculate the genetic relatedness between pairs of individuals in the space of N SNPs [3, 20]. We define the following GRM distance matrix between individual i and j,

10 $$ {D}_{ij}^{GRM}=\sqrt{2N\left(1-{A}_{ij}\right)}, $$

where

11 $$ {A}_{i j}=\frac{1}{N}\sum \limits_{\nu =1}^N\frac{\left({g}_{i\nu}-2{p}_{\nu}\right)\left({g}_{j\nu}-2{\mathrm{p}}_{\nu}\right)}{2{p}_{\nu}\left(1-{p}_{\nu}\right)} $$

and *g*_*iν*_ is the number of copies of the reference allele for the *ν*^*th*^ SNP of the *i*^*th*^ individual and *p*_*ν*_ is the frequency of the reference allele [3]. Each genotype in the summand is standardized by *p*_*ν*_ to account for the differences in allele frequency between SNPs. In addition to comparing diff functions (Eqs. 4, 5, 8) in the attribute importance score, we also compare Manhattan (Eq. 9) and GRM (Eq. 10) methods for computing the pairwise distances to find nearest neighbors.

### Non Relief-based comparison methods: Random Forest, lasso, and random gene

Random Forests (RF) is a widely used machine learning classifier and feature selector that grows an ensemble of classification trees in bagged samples with random attribute selection [21]. To measure the importance of a feature after training, the values of that feature are permuted among the training data and the out-of-bag error is again computed on this perturbed data set. The importance score for the feature is computed by averaging the difference in out-of-bag error before and after the permutation over all trees and the score is normalized by the standard deviation of these differences [4, 22–24]. We used the “Ranger” implementation of Random Forest, which is included in our inbix software to compute classification and variable importance.

We used the PLINK software to perform Lasso (--lasso). We adjusted for the first five principal components, and the PCs were not subject to model selection. The top Lasso variants were chosen by top regression coefficients. We used h^2^ = 0.5 as the estimate of the heritability to calibrate the regression and we used *λ*_min_ = 0.001 for the L1 penalty parameter. Finally, we generated lists of random genes as a null comparison list that shows the effect of pathway size on enrichment. A random set of 500 genes is randomly sampled 100 times and the average overlap of the 500 genes is computed for each pathway. Estimating the expected number of overlapping genes by chance helps to calibrate the overlaps of each pathway for the non-random feature selection methods.

#### ReliefF software implementation

We performed all preprocessing and ReliefF analyses using our **I**nteraction-**N**etwork **BI**onformatics Toolbo**X** (**inbix**) software for machine learning and epistasis network analysis for high-dimensional data. Inbix is a free, open-source, command-line bioinformatics tool, written in C++ and designed to perform a range of large-scale analyses with computational efficiency. The source is publicly available from our website and github (http://insilico.utulsa.edu/index.php/inbix/ and https://github.com/insilico/inbix) [18, 25, 26]. The inbix tool supports the PLINK format and includes PLINK algorithms and utilities [27] along with new machine learning and network analysis methods. In our inbix software, we use the following command to execute ReliefF with a GWAS binary bed file <bed-file>.bed and a file containing phenotype information <pheno-file>.pheno:

- ./inbix --bfile <bed-file> --relieff --pheno <pheno-file> --snp-metric-nn <nn-metric> --snp-metric-diff <diff-metric> --out <results-file>
  where <diff-metric> can take on values gm, am, or ti/tv and <nn-metric> can also take on values gm, am, or titv with manhattan or Euclidean for combining the individual SNP diffs. The user may alternatively select grm in <nn-metric>, which uses GRM as the nearest-neighbor metric and does not use the diffs. Any combination of <diff-metric> or <nn-metric> is allowed. For the MDD GWAS with 922 individuals and 281,648 SNPs (after filtering), the GRM metric takes approximately 8 hours and the other metrics take approximately 12 hours of CPU time (additional details in the Additional file 1).
  We use the constant-k ReliefF algorithm in inbix with the diffs and metrics described above. With inbix it is possible to optimize the number of neighbors for each attribute [18]. However, for this study, we use the constant value, *k* = ⌊*m*/6⌋, for ReliefF nearest neighbors, where m is the number of samples. The value *k* = ⌊*m*/6⌋ is the inbix default and was chosen based on Ref. [28] where it was shown to approximate the adaptive radius Relief method, MultiSURF [29], which balances power to detect epistatic effects and main effects.

### GWAS data, filtering and mapping

In this study we compare ReliefF metrics with each other and with other analysis methods based on enrichment of selected features in functional pathways for MDD. We used a recent GWAS of MDD [30] that includes 922 European individuals that were recruited through a survey of 1259 individuals who filled out forms and telephone interviewed for DSM-IV covering depressive, bipolar, psychotic, alcohol, substance and anxiety disorders as well as family history of mood disorders. After exclusions, extracted DNA was genotyped with the Illumina Omni1-Quad microarray for 463 cases of MDD and 459 controls. For all ReliefF analyses, we used constant *k* = ⌊*m*/6⌋ = 138 nearest neighbors.

#### Filtering and mapping of genes and pathways

Additional details of data processing and analysis, including command line scripts, is provided in the Additional file 1. In the initial steps of analysis, dimensionality reduction is performed on SNPs by linkage disequilibrium (LD) with threshold 0.5 and minor allele frequency threshold 0.01. The goal of this filtering is to remove highly redundant and very low signal data as well as obtain a manageable number of variants for machine learning analysis (we obtain 281,648 variants).

A list of ranked SNPs for each method is obtained from the algorithms to be compared (Fig. 1 method overview). We use Ensembl IDs to map the top SNPs to genes [31]. Given the rs-number of a SNP, the algorithm finds the location of the variant relative to genes, and the SNP is mapped to the gene symbol of the closest gene [32]. In the supplement, we include a link to our webservice that accepts a list of SNP rs-numbers and returns mapped genes to a table, and we include the R code for the mapping. Despite LD pruning, many top SNPs will map to the same gene; thus, we begin the mapping with more than 500 top SNPs so that we end up with 500 top unique genes.

We then use Molecular Signatures Database (Msigdb) [33] to identify the number of genes in our top 500 gene selection lists that overlap with target pathways. The overlaps are based on HUGO gene symbols. Our goal is not to compute the statistical significance of overlap for discovery, but to compare the number of genes found in known pathways relative to other gene ranking methods. Thus, the size of the gene background is not a concern. We also use random gene selection, mentioned above, to show the expected amount of overlap with a pathway by chance.

## Results

**Fig. 5:**
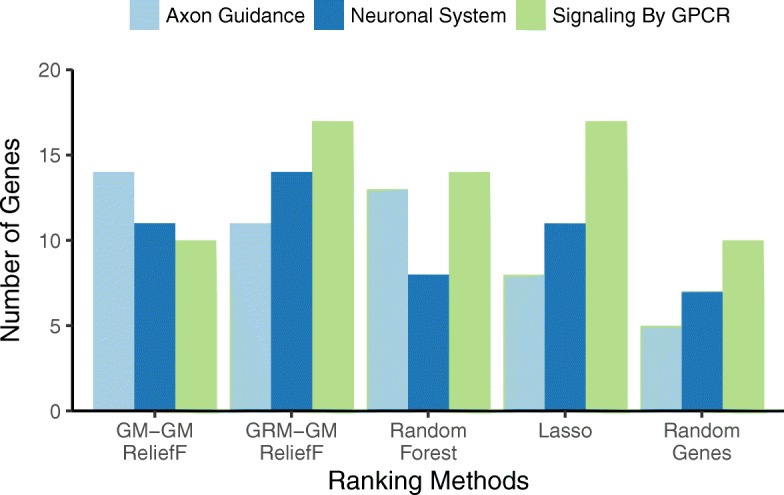
Pathway detection comparison of standard ReliefF (GM-GM) and ReliefF with GRM nearest neighbors (GRM-GM). Bars count the number of genes that overlap the given pathway – Axon Guidance (light blue), Neuronal System (dark blue), and G protein-coupled receptor (GPCR) (green) – from the top 500 genes from each feature selection method. The methods are GM-GM (standard ReliefF with GM-based nearest neighbors (Eqs. 4 and 9) and GM attribute diff (Eq. 4)), GRM-GM (ReliefF with GRM-based nearest neighbors (Eq. 10) and standard GM attribute diff (Eq. 4)), where GM is genotype mismatch and GRM is genetic relationship metric. In addition to ReliefF methods, we compare with Lasso corrected for principal components, Random Forest, and Random Genes (random sampling of 500 genes averaged over 100 replicates). GRM-GM detects more genes than other methods with the exception of GM-GM and Random Forest finding more Axon genes

**Fig. 6:**
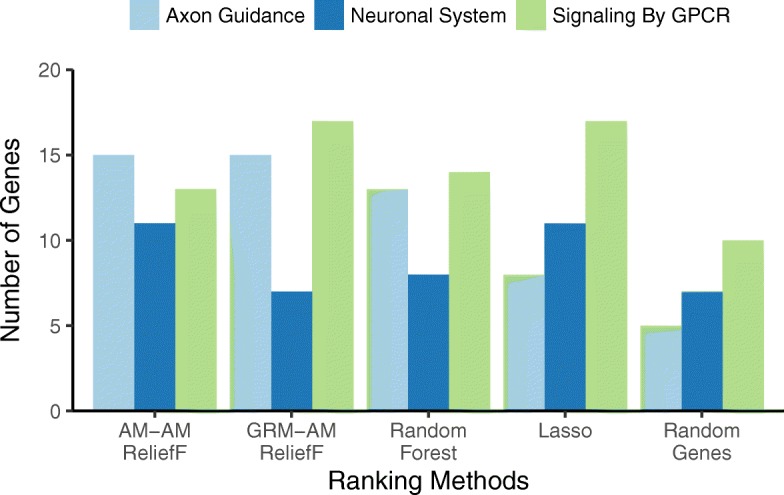
Pathway detection comparison of AM-AM ReliefF and GRM-AM ReliefF. Bars count the number of genes that overlap the given pathway – Axon Guidance (light blue), Neuronal System (dark blue), and G protein-coupled receptor (GPCR) (green) – from the top 500 genes from each feature selection method. The methods are AM-AM (ReliefF with AM-based nearest neighbors (Eqs. 5 and 9) and AM attribute diff (Eq. 5)), GRM-AM (ReliefF with GRM-based nearest neighbors (Eq. 10) and AM attribute diff (Eq. 5)), where AM is allele mismatch and GRM is genetic relationship metric. In addition to ReliefF methods, we compare with Lasso with principal component covariates, Random Forest, and Random Genes (random sampling of 500 genes averaged over 100 replicates). AM-AM and GRM-AM have a similar pattern to and better performance than Random Forest. GRM-AM finds the most Axon and GPCR genes, and AM-AM finds the most Neuronal genes

**Fig. 7:**
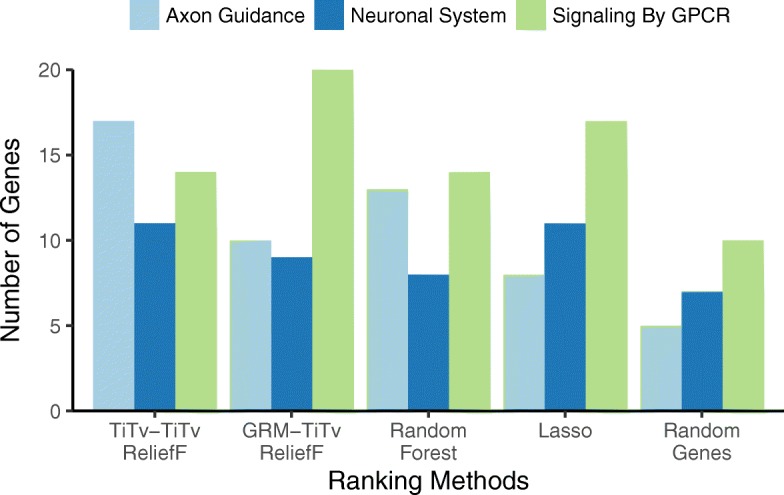
Pathway detection comparison of TiTv-TiTv ReliefF and GRM-TiTv ReliefF. Bars count the number of genes that overlap the given pathway – Axon Guidance (light blue), Neuronal System (dark blue), and G protein-coupled receptor (GPCR) (green) – from the top 500 genes from each feature selection method. The methods are TiTv-TiTv (ReliefF with TiTv-based nearest neighbors (Eqs. 8 and 9) and TiTv attribute diff (Eq. 8)), GRM-TiTv (ReliefF with GRM-based nearest neighbors (Eq. 10) and TITv attribute diff (Eq. 8)), where TiTv is transition/transversion diff and GRM is genetic relationship metric. In addition to ReliefF methods, we compare with Lasso with principal component covariates, Random Forest, and Random Genes (random sampling of 500 genes averaged over 100 replicates). TiTv-TiTv has a similar pattern to but better performance than Random Forest. GRM-TiTv finds the most GPCR genes, and TiTv-TiTv finds the most Axon and Neuronal genes

We evaluate feature selection methods based on the number of genes found in pathways that have been implicated in mood disorders (Figs. 5, 6 and 7). We chose pathways related to G protein-coupled receptors (GPCRs) because they are implicated in pathophysiology of MDD as well as bipolar disorder [34]. For example, current pharmacological interventions for MDD target neuromodulators (serotonin, norepinephrine, dopamine) that signal via GPCR systems. The other two pathways, Axon Guidance and Neuronal System, have been hypothesized to play an important role in mood disorder pathophysiology. In addition to ReliefF metrics, we compare with feature selection by Lasso, Random Forest, and the average of random gene selection to assess the number of genes expected by chance due to pathway size.

We compare combinations of ReliefF attribute diff functions for use in the average miss and hit calculations (Eqs. 2 and 3) and metrics for computing nearest neighbors in the full space of SNPs. The diff functions are GM, AM, and Ti/Tv (Eqs. 4, 5, and 8) and nearest neighbor metrics are the Manhattan metric (Eq. 9) with the GM, AM and Ti/Tv diffs and the GRM metric (Eq. 10). We compare the three combinations of <diff-metric> and two possible <nn-metric> for a total of 3x2=6 combinations (Figs. 5, 6 and 7). We combine the three diffs with the same diff in a Manhattan metric to form the first three “<nn-diff>-<attribute-diff>” combinations: GM-GM, AM-AM, TiTv-TiTv. We also combine the three diffs with GRM nearest-neighbor metric to form the other three “<GRM-nn>-<attribute-diff>” combinations: GRM-GM, GRM-AM, GRM-TiTv.

We first note that for random gene selection (“Random Genes” on right-most side of Figs. 5, 6 and 7), the pathway overlap is correlated with the size of each pathway. As expected, choosing random genes will result in a certain amount of overlap with a pathway by chance in proportion to the size of the pathway. All methods perform better than chance (“Random Genes”) for detecting all pathways. For the Axon Guidance pathway, TiTv-TiTv (Eq. 8 for the Manhattan metric and attribute diff) detected the most genes (light blue, Fig. 7). For Signaling by GPCR pathway, GRM-TiTv (GRM nearest neighbor metric and TiTv attribute diff) detected the most genes (green, Fig. 7). For Neuronal System, GRM-GM performed best (dark blue, Fig. 5).

Increasing in complexity of the diff (GM, AM, TiTv in Figs. 5, 6 and 7, respectively), shows pathway enrichment increasing when the attribute diff is used in the Manhattan metric (GM-GM < AM-AM < TiTv-TiTv). This suggests a benefit to including transition/transversion information in the attribute diff calculation for attribute importance. When the diffs are combined with GRM, the GPCR pathway enrichment increases significantly. The GRM metric adjusts for heterogeneity of allele frequencies, and detecting genes that contain SNPs with such heterogeneity likely benefits from GRM.

## Discussion and conclusions

Machine learning feature selection is a powerful tool for discovery in large data like GWAS with complex population and interaction structure. ReliefF is particularly efficient and powerful at selecting genes that are enriched for gene-gene interactions. However, in past studies, the diff function used to compute nearest neighbors and for updating attribute importance has treated information about SNPs simplistically. Thus, we introduced more complex and genetically relevant mathematical functions for computing diffs, including the development of a new 2d transition/transversion genotype encoding and associated diff. To find nearest neighbors, ReliefF implementations typically use a Manhattan metric of the attribute diffs. Euclidean can also be used to combine diffs, but past results have indicated little difference with Manhattan. A Mahalanobis distance function has also been developed, which allows for non-spherical neighborhoods [35]. In addition to Manhattan with the new diff functions, we used the genetic relationship matrix (GRM) to compute nearest-neighbor distances, which has not been used previously in ReliefF.

When testing for pairwise epistatic effects in a linear model, one may decompose the epistatic effects into additive and dominant encodings [36]. ReliefF has less flexibility to mix encodings than a pairwise-SNP linear model; however, ReliefF ranks SNPs within the context of all other SNPs in the dataset, which may include pairwise and higher-order interactions. In our approach, we are able to mix encodings in a given ReliefF analysis by using different diff functions for attribute importance (Eqs. 1–3) and for finding nearest neighbors (Eqs. 9 or 10). The GM diff is based on a dominant single-locus encoding and the AM diff is based on an additive encoding. The Ti/Tv diff is based on a new 2d Ti/Tv encoding where a genotype is mapped onto a unit sphere and contracts transition genotypes closer together than corresponding transversion genotypes (Fig. 4).

For each method, the top 500 SNPs were mapped to genes and overlap with the relevant biological pathways for major depressive disorder (MDD) was calculated. Our results provide evidence that using either AM (Eq. 5) or Ti/Tv (Eq. 8) diffs in the attribute importance score calculation (Eqs. 1–3) has an advantage over the simple GM diff (Eq. 4). The detection of genes in certain pathways also can be improved by combining the attribute diffs with the GRM metric (Eq. 10) for computing nearest neighbors.

The GRM method for finding nearest neighbors has the useful property of adjusting for alleleic heterogeneity. Using GRM to compute nearest neighbors results in the best enrichment for two of the three pathways: GPCR Signaling with GRM-TiTv (green, Fig. 7, GRM nearest neighbor metric and TiTv attribute diff) and Neuronal System with GRM-GM (dark blue, Fig. 5). The 2d TiTv encoding is the same as the AM diff for transversion SNPs but results in genotype differences that are contracted closer together for transition SNPs. Using TiTv results in the best enrichment for the third pathway: Axon Guidance with TiTv-TiTv (light blue, Fig. 7, TiTv used for the nearest neighbor metric diff and attribute diff).

We focused on the analysis of real data as a proof of principle. Additional insight may be obtained by using a simulation strategy that incorporates transition and transversion differences that affect the phenotype. The challenge is to make such a simulation biologically realistic and not artificially biased toward one method. We used ReliefF’s simple nearest neighbor-finding method with *k* = ⌊*m*/6⌋ because it balances the ability to find main effect and interaction effects [28]. However, there are other Relief-based methods that may be used to optimize genetic findings. For example, one may use an adaptive attribute-specific number of neighbors to improve power to detect main effects and interaction effects [18]. One may also increase power through adaptive radius versions of Relief, like SURF [37], MultiSURF [29], and STIR [28], or through backwards elimination versions of Relief, like iterative ReliefF.

The ReliefF methods generally perform better than the non-ReliefF methods (Random Forest and Lasso). However, we note that no single analysis method can extract all information from a whole-genome association study (i.e., no free lunch) and each method finds unique gene signatures that can contribute to the overall picture of the pathway or phenotype. Thus, combining metrics and feature selection methods may be a good strategy for maximizing the detection of relevant genes. There are opportunities to improve these methods by incorporating additional information about the biological properties of the data, similar to phylogenetic substitution matrices or using coding/non-coding information. One may also assess the false discovery rate of different metrics using the STIR approach for determining the statistical significance of Relief scores [28].

## Additional file

Additional file 1: Command line details to use inbix to perform GWAS filtering and pathway enrichment and feature selection by random forest, lasso, and ReliefF with new transition-transversion diff and genetic relationship metric. (DOCX 35 kb)
