# Supplementary material for: Transition-transversion encoding and genetic relationship metric in ReliefF feature selection improves pathway enrichment in GWAS
Source: BioData Min. 2018 Nov 3;11:23. doi: 10.1186/s13040-018-0186-4 (PMC6215626; doi:10.1186/s13040-018-0186-4)
Supplement: Supplementary file 1 — Command line details to use inbix to perform GWAS filtering and pathway enrichment and feature selection by random forest, lasso, and ReliefF with new transition-transversion diff and genetic relationship metric. (DOCX 35 kb) [file 13040_2018_186_MOESM1_ESM.docx]

**Additional file 1**

“Transition-transversion encoding and genetic relationship metric in ReliefF feature selection improves pathway enrichment in GWAS”

# GWAS data Analysis

We performed all preprocessing and analyses using the **I**nteraction-**N**etwork **BI**onformatics Toolbo**X** (**inbix**) software for machine learning and epistasis network analysis for high-dimensional data, including GWAS, microarray, RNA-Seq, eQTL, and others. The inbix software is a free, open-source, command-line bioinformatics tool, written in C++ and designed to perform a range of large-scale analyses with computational efficiency.

<http://insilico.utulsa.edu/index.php/inbix/>

<https://github.com/insilico/inbix>

Inbix includes Relief-based and evaporative cooling-based algorithms for feature selection for detecting main effects and interaction effects for case-control and quantitative trait data. Inbix also allows epistasis and expression-epistasis network inference for GWAS and gene expression data; epistasis network centrality analysis; differential co-expression network analysis; interaction QTL (iQTL) network inference and differential-coexpression Variant (dcVar) analysis of eQTL data.

The initial LD and minor allele frequency pruning was done with inbix options:

./inbix --noweb --bfile file --maf 0.01 --indep-pairwise 50 5 0.5 --out output

./inbix --noweb --bfile file --extract filename.prune.in --make-bed --out output

After preprocessing, 281,648 SNPs remain for feature selection. Some of the machine learning algorithms are run on desktop computers and others on a supercomputer. Below is a sample of the batch script followed by different analysis commands submitted to the supercomputer for this study:

#!/bin/bash

#SBATCH --partition=normal (the name of the partition to be used)

#SBATCH –exclusive (request exclusive access on the participating compute nodes, so that other batch jobs don't run on the same compute nodes as this batch job, and therefore don't interfere with it)

#SBATCH -c 20 (number of cores)

#SBATCH --output=jobname_%J_stdout.txt,

#SBATCH --error=jobname_%J_stderr.txt (tells SLURM to send ouput and error messages to the filenames listed)

#SBATCH --job-name=jobname (name of the batch job)

#SBATCH --mail-user=youremailaddress@yourinstitution.edu (the e-mail address to send notifications to)

#SBATCH --mail-type=ALL (e-mail a notification when the batch job either completes or fails)

#SBATCH --chdir=/home/yourusername/directory_to_run_in

if [ -n "$SLURM_CPUS_PER_TASK" ]; then

omp_threads=$SLURM_CPUS_PER_TASK

else

omp_threads=1

fi

export OMP_NUM_THREADS="$omp_threads”

echo "Number of parallel processes: $OMP_NUM_THREADS"

echo "Number of slurm: $SLURM_CPUS_PER_TASK"

# Methods

## Relief-F feature selection method

In this step, we show relevant inbix options, such as --snp-metric-nn (metric for determining the distance between subjects (gm|am|titv|grm), --snp-metric-diff (metric for determining the diff(erence) between subjects for one SNP (gm|am|titv), --k-nearest-neighbors (we use the default k nearest neighbors, k=floor((m-1)*0.15)), --pheno (specifices the phenotype file).

./inbix --bfile file --relieff --pheno filename.pheno --snp-metric-nn gm|am|titv|grm --snp-metric-diff gm|am|titv --out outputFileName

The output file from this process is a text file with prefix outputFileName and “.relieff.tab” extension that contains two columns in which the first column shows the rs numbers and the second column indicate the weight of each SNP. Inbix also supports computing the statistical significance of ReliefF scores.

The table below shows the computation time for each combination of diff and metric used in ReliefF algorithm:

| **Metric Combinations** | **Time** |
| --- | --- |
| GM-GM | 12 hr and 16 min |
| GM-AM | 12 hr and 18 min |
| GM-TiTv | 12 hr and 32 min |
| AM-GM | 12 hr and 15 min |
| AM-AM | 12 hr and 21 min |
| AM-TiTv | 12 hr and 33 min |
| GRM-GM | 7 hr and 58 min |
| GRM-AM | 7 hr and 58 min |
| GRM-TiTv | 8 hr and 15 min |
| TiTv-TiTv | 12 hr and 40 min |

### Transition/Transversion Ratio Calculation

1) awk -F ' ' 'FNR<=500{print $2}' name.relieff.tab > name_top500.txt (get top 500 SNPs from relieff file that contains two columns and save it into a .txt file)

2) ./plink --bfile MDD_LD --extract name_top500.txt --make-bed --out outputFileName(get the bed file of the top 500 SNPs)

3) ./plink --bfile outputFileName --recode vcf --out outputFileName (get the vcf file)

4) vcftools --vcf outputFileName.vcf --TsTv-summary (get Ti/tv ratio and count)

## Random Forest method

In this method Ranger software has been used:

1) ./inbix --bfile file --recodeA --out SNPs (this step converts the binary file)

2) cat SNPs.raw | cut –d ' ' –F 7 -> Snps (This step gets the seventh row of the file, rs numbers and save all of them into a new file)

3) cat SNPs.raw | cut –d ' ' –F 6 -> pheno ( get the pheno file)

4) paste Snps pheno > newdataset.txt

5)./ranger --verbose --file filename --depvarname PHENOTYPE --treetype 1 --impmeasure 2 --ntree 500 --nthreads 4 --write --out filename.train

We set the number of trees to 500 and set the tree type to classification and set importance mode to “Permutation importance, scaled by standard errors” type and use the default values for other parameters as provided by the program.

## Principle Component Analysis

./inbix --bfile filename_LD --genome --out filename #(get .genome file)

./inbix --bfile filename_LD --cluster --pca header --extract filename.prune.in --read-genome filename_LD.genome #(get plink.eigenval and plink.eigenvec & Cluster solution written to plink.cluster1 , plink.cluster2 , and plink.cluster3)

./inbix --bfile filename_LD --pheno filename.pheno --allow-no-sex --covar plink.eigenvec --covar-name PC1,PC2,PC3,PC4,PC5 --out association --logistic -–adjust #(do the association analysis )

## Least Absolute Shrinkage and Selection Operator (LASSO) Association Analysis:

./inbix --bfile filename_LD --pheno filename.pheno --allow-no-sex --covar plink.eigenvec --covar-name PC1,PC2,PC3,PC4,PC5 --out association --lasso 0.5 0.01 --adjust

# Mapping SNPs to Genes

We mapped top ranked SNPs to their corresponding genes using the shiny application, which uses the R code below.

http://129.244.244.104:3838/insilico-gene-annotation-app/

The following code uses a package called "BiomaRt" to help map the SNPs to genes. It first gets the rs-number and then finds its corresponding Ensembl gene and transcript ID and then maps the ID to the gene symbol.

library(data.table)

library(biomaRt)

library(openxlsx)

source("https://bioconductor.org/biocLite.R")

biocLite("clusterProfiler")

snps.file <- "snp1.txt"

# lookup the refsnp IDs in the “snp1.txt” file and return the ENSEMBL IDs

the.snps <- read.table(snps.file, header=FALSE, stringsAsFactors=FALSE)[, 1]

# load the SNP info database

snp2ensembl.biomart<- useMart("ENSEMBL_MART_SNP", dataset="hsapiens_snp")

snp.id.info <- getBM(c("refsnp_id", "ensembl_gene_stable_id", "ensembl_transcript_stable_id",

"chr_name", "chrom_start", "chrom_end"),

filters="snp_filter",

values=the.snps,

uniqueRows=FALSE,

mart=snp2ensembl.biomart)

map.ensembl.clean <- snp.id.info[snp.id.info$ensembl_gene_stable_id != "", ]

#uniq.ensembl <- snp.id.info[which(snp.id.info$ensembl_gene_stable_id %in% unique(snp.id.info$ensembl_gene_stable_id)), ]

# lookup the ENSEMBL ID and get the gene symbol

ensembl2gene.biomart <- useEnsembl(biomart="ensembl", dataset="hsapiens_gene_ensembl")

snp.gene.info <- getBM(attributes=c('ensembl_gene_id', 'hgnc_symbol'),

filters='ensembl_transcript_id',

values=map.ensembl.clean$ensembl_transcript_stable_id,

uniqueRows=TRUE,

mart=ensembl2gene.biomart)

snp.genes.annot <- cbind(map.ensembl.clean[match(snp.gene.info$ensembl_gene_id, map.ensembl.clean$ensembl_gene_stable_id), 1],

snp.gene.info)

colnames(snp.genes.annot) <- c("RefSNP", "Ensembl", "Gene")

snp.genes.annot <- snp.genes.annot[snp.genes.annot$Gene != "", ]

snp.genes.annot[order(snp.genes.annot$RefSNP, snp.genes.annot$Ensembl), ]

gene.symbols <-snp.genes.annot[,3]

# Pathway Analysis

We obtained Reactome pathways from the Msigdb website, <http://software.broadinstitute.org/gsea/msigdb/index.jsp>, and we wrote the Python code below to get gene sets for a list of genes.

import sys

from collections import defaultdict

def parseMsigGmtFile(genesetFilename):

print "Reading MsigDB gene set: " + genesetFilename

gsFile = open(genesetFilename)

geneset = {}

for line in gsFile:

lineParts = line.strip().split()

genesetKey = lineParts[0]

genesetDesc = lineParts[1]

genesetGenes = lineParts[2:]

geneset[genesetKey] = genesetGenes

gsFile.close()

print "Read " + str(len(geneset)) + " gene sets"

return geneset

def showMsigGeneSets(genesFilename, genesetFilename, topN, outputFilename):

geneset = parseMsigGmtFile(genesetFilename)

genesFile = open(genesFilename)

myGenes = []

for line in genesFile:

myGenes.append(line.strip())

genesFile.close()

overlap = defaultdict(list)

for genesetKey in geneset:

for gene in geneset[genesetKey]:

if gene in myGenes:

overlap[genesetKey].append(gene)

print "Writing output file: " + outputFilename

outFile = open(outputFilename, "w")

topCounter = 0

for geneset in sorted(overlap.items(), key=lambda x: len(x[1]), reverse=True):

if topCounter < topN:

outFile.write(geneset[0] + "\t")

outFile.write(str(len(overlap[geneset[0]])) + "\t")

outFile.write(",".join(overlap[geneset[0]]) + "\n")

topCounter += 1

else:

continue

outFile.close()

if __name__ == "__main__":

if len(sys.argv) < 5:

print "Usage: " + sys.argv[0] + " <gene list file> <MSig gene set file> <topN> <output file>"

sys.exit()

showMsigGeneSets(sys.argv[1], sys.argv[2], int(sys.argv[3]), sys.argv[4])
